# Comparison of Injectable Biphasic Calcium Phosphate and a Bovine Xenograft in Socket Preservation: Qualitative and Quantitative Histologic Study in Humans

**DOI:** 10.3390/ijms23052539

**Published:** 2022-02-25

**Authors:** Marija Čandrlić, Matej Tomas, Matej Karl, Lucija Malešić, Aleksandar Včev, Željka Perić Kačarević, Marko Matijević

**Affiliations:** 1 Department of Dental Medicine, Faculty of Dental Medicine and Health Osijek, J. J. Strossmayer University of Osijek, 31000 Osijek, Croatia; marija.candrlic@fdmz.hr (M.Č.); matej.tomas@fdmz.hr (M.T.); karlmatej@gmail.com (M.K.); 2 Faculty of Medicine Osijek, J. J. Strossmayer University of Osijek, 31000 Osijek, Croatia; 3 Private Dental Practice Matej Karl, Prilaz Vilka Ivekovića 1, 31511 Belišće, Croatia; 4 Department of Anatomy, Faculty of Medicine, University of Rijeka, 51000 Rijeka, Croatia; lucija.malesic@gmail.com; 5 Department of Pathophysiology, Physiology and Immunology, Faculty of Dental medicine and Health Osijek, J. J. Strossmayer University of Osijek, 31000 Osijek, Croatia; aleksandar.vcev@fdmz.hr; 6 Department of Anatomy, Histology, Embriology, Pathology Anatomy and Pathology Histology, Faculty of Dental Medicine and Health Osijek, J. J. Strossmayer University of Osijek, 31000 Osijek, Croatia; 7 Community Healthcare Center of Osijek-Baranja County, 31000 Osijek, Croatia

**Keywords:** socket preservation, guided bone regeneration, biphasic calcium phosphate, xenograft, tissue engineering, qualitative histology, quantitative histology

## Abstract

This study is the first histologic evaluation of an injectable biphasic calcium phosphate (IBCP) in humans six months after socket preservation according to the principles of guided bone regeneration. After tooth extraction, the alveolar ridge of 21 patients was augmented with IBCP (maxresorb^®^ inject) in the test group, while 20 patients in the control group received a bovine xenograft (BX) (cerabone^®^). Six months after augmentation, a reentry procedure was performed to collect biopsies of regenerated bone for qualitative and quantitative histologic analysis. A total of 20 biopsies were taken for analysis. Qualitative histologic analysis showed complete integration of the biomaterial and no inflammatory tissue reaction, indicating the biocompatibility of the bone grafts and the surrounding tissue in both groups. Histomorphometric analysis showed comparable results in terms of newly formed bone (IBCP: 26.47 ± 14.71%, BX: 30.47 ± 16.39%) and residual biomaterial (IBCP: 13.1 ± 14.07%, BX: 17.89 ± 11.81%), with no significant difference found across groups (*p* > 0.05, Mann—Whitney U test). Statistical significance between groups was found in the result of soft tissue percentage (IBCP: 60.43 ± 12.73%, BX: 51.64 ± 14.63%, *p* = 0.046, Mann—Whitney U test). To conclude, IBCP and BX showed good osteoconductivity and biocompatibility with comparable new bone formation six months after alveolar ridge preservation.

## 1. Introduction

For optimal functioning of the entire stomatognathic system, any lost functional tooth units must be replaced. It is known that dental implants are considered the most suitable and comfortable therapeutic option for the replacement of one or more missing teeth [1]. Unfortunately, bone remodeling after tooth extraction does not usually result in restitutio at integrum, which would be ideal for a dental implant placement. Most dimensional changes occur in the first three months after healing, but bone remodelling lasts up to one to three years after extraction [2,3]. Differences in the patient’s bone biology, the size of the alveolar defect, bone trauma during extraction, and the presence of bone fenestration or/and dehiscences also affect bone remodeling [4]. To minimize all of the above difficulties, it is recommended to perform one of the augmentation procedures before implant placement. The purpose of augmentation is to create a skeleton that supports new bone formation. Guided bone regeneration (GBR) is a reliable and acclaimed method to achieve bone growth. The basis of GBR is the isolation of the bone defect by a physical membrane against the soft tissue to support the growth of the slow-moving osteogenetic cells [5,6]. However, the success of GBR and implant-prosthetic rehabilitation depends not only on therapeutic clinical skills, but also on the proper selection and handling of bone grafting material. It is well known that the gold standard among bone grafts is autogenous bone, but due to complications in its use and limited availability, work is being done to develop alternatives [7,8]. Therefore, allografts, xenografts, and alloplasts have emerged as promising alternatives to autogenous bone.

Xenografts are bone substitutes of animal origin, usually from cattle, pigs, or horses. Before use, a graft taken from an animal must undergo a mechanical and chemical purification process to remove the organic component. The result of the purification process is hydroxyapatite granules, which are very similar to hydroxyapatite in human bone [9]. Clinical, histological, and radiological studies have confirmed the biocompatibility and safety of xenografts as well as their osteoconductive properties [10,11,12,13,14,15,16]. Jensen et al. [17] described a histological integration of inorganic bovine bone granules with new bone. Hydroxyapatite granules were shown to remain permanently integrated into the structure of regenerated new bone after healing was complete, forming a bone composite with better mechanical and physical properties [17]. Theoretically, there is a potential risk of transmission of prion infection when using bovine xenografts (BX). However, studies have shown that the risk is negligible, especially nowadays when purification procedures for animal transplants are very advanced [9]. Ethical and religious reasons are also mentioned in the literature as a common reason why some patients reject xenografts [18,19]. Due to their good mechanical properties and resorption strength, xenografts are often used in combination with autogenous bone to achieve better volume stability of the augmented area [20]. They are available in various forms, most commonly as granules that need to be mixed with saline or blood before use, or as prefabricated blocks that are attached to the defect site with pins [21,22].

Biomimetic materials are founded on the fundamental pathways of biomineralization. Understanding the nanostructure of bone and the organic-inorganic interactions of the bone matrix is essential for developing biomaterials that resemble natural bone. These interactions are based on the affinity of the cationic functional groups for the calcium and phosphate ions of the bone mineral. The ultimate goal is to achieve the ultrastructure and morphology of natural biominerals [23,24]. Alloplastic grafts for dental purposes are usually based on hydroxyapatite (HA), beta-tricalcium phosphate (β-TCP) and alpha-tricalcium phosphate (α-TCP) and combinations thereof. Biphasic calcium phosphate (BCP) was formed by the fusion of HA and β-TCP in various ratios, most commonly 60:40 and 70:30 [25]. Our focus is on injectable biphasic calcium phosphate (IBCP). Histologic studies in animal models have shown that injectable bone substitutes have good osteoconductive properties and that their use results in satisfactory new bone formation [26,27,28,29,30]. Papanchev et al. [31] published a case report comparing the percentage of new bone formation at two healing time points after sinus floor augmentation. Our group of authors has previously published a case report on the histologic and radiologic results of a four-phase injectable synthetic bone graft in GBR [32]. An extensive literature search revealed that no randomization clinical trial to date has evaluated the use of IBCP, composed of water-based gel with nano-hydroxyapatite particles and biphasic granules (60% HA and 40% β-TCP) for the indication of alveolar ridge preservation according to GBR principles. Hence, the aim of the presented randomized clinical trial was to evaluate and compare the safety and efficacy of the above-mentioned IBCP and a BX in alveolar ridge preservation using qualitative and quantitative histologic analysis.

## 2. Materials and Methods

### 2.1. Preoperative Assessment and Protocols

The study was conducted in accordance with the Declaration of Helsinki and approved by the Ethics Committee of the Community Healthcare Center of Osijek-Baranja County, Croatia (No. 03-1897/20) and the Ethics Committee of the Faculty of Medicine in Osijek (Class: 2158-61-07-21-18, No. 602-04/21-08/07). 

**Figure 1:**
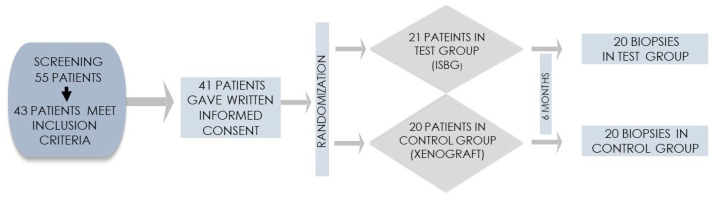
Flowchart of the randomization process.

Patients were recruited in an oral surgery office in Osijek, Croatia. Before participating in the study, they were informed in detail about the study protocol and gave their written informed consent. A total of 55 patients were screened, of whom 43 met the inclusion criteria and 41 finally gave written informed consent to participate in this clinical trial (Figure 1).

Patients had to be at least 18 years old, indicated for at least one tooth extraction and later for implant-supported rehabilitation. The inclusion criteria were: age over 18 years, good physical and mental health, and patients’ willingness to undergo regular follow-up examinations. Patients with systemic diseases (e.g., patients with uncontrolled cardiovascular or endocrine disease, osteoporosis, or immunosuppressed patients) or who received radiation therapy, glucocorticoid or bisphosphonate therapy, and pregnant or lactating women were excluded. In addition, the following local exclusion criteria applied: smoking (>10 cigarettes per day), acute infection at the extraction site, untreated periodontal disease, or poor oral hygiene [33,34].

Patients who gave written informed consent underwent supra- and sub-gingival removal of soft and hard plaque and were instructed to maintain adequate oral hygiene. One hour before the procedure, both groups received a single dose of oral amoxicillin with clavulanic acid (Klavocin^®^ 875 mg + 125 mg, Pliva, Zagreb, Croatia) or oral clindamycin (Klindamicin-MIP^®^ 600 mg, MIP Pharma Croatia, Zagreb, Croatia) in case of penicillin hypersensitivity. Patients rinsed their mouths with chlorhexidine (PerioPlus^®^ 0.2%, Curaprox, Flawil, Switzerland) before administration of anesthesia.

### 2.2. Surgical and Postoperative Protocol

**Figure 2:**
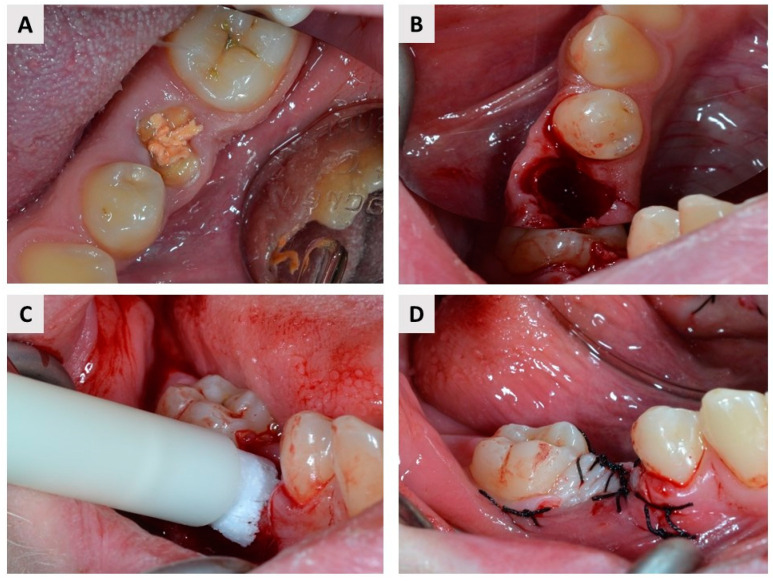
(**A**) Occlusal photograph of a root without a crown (lat. *radix relicta*) that was predisposed to extraction. (**B**) Alveolar socket following atraumatic extraction and curettage. (**C**) Application of maxresorb^®^ inject. (**D**) Complete coverage of the biomaterials (IBCP and resorbable membrane) was achieved with single 5/0 sutures.

**Figure 3:**
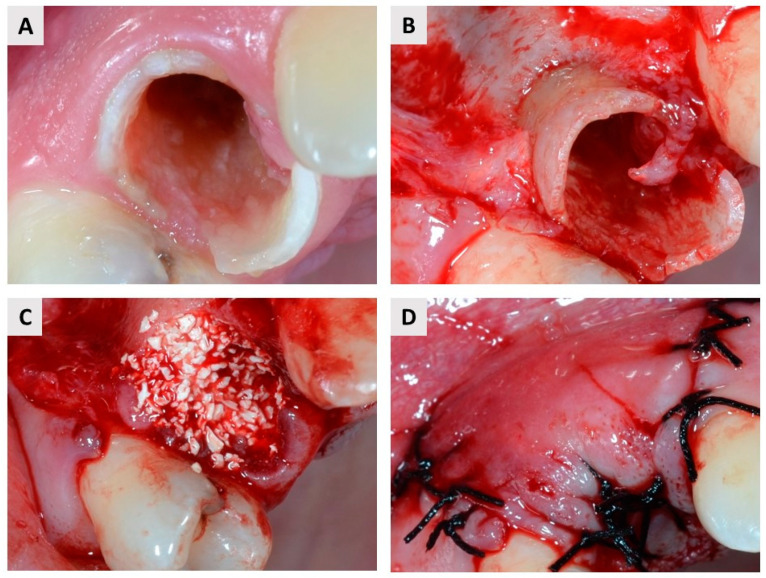
(**A**) Occlusal image of a hopeless premolar. (**B**) The mucoperiosteal flap was elevated before tooth extraction. (**C**) Application of the cerabone^®^. (**D**) Complete coverage of the biomaterials (BX and resorbable membrane) was achieved with single 5/0 sutures.

The patients underwent the surgery under local anesthesia (Lidokain Belupo^®^ 2%, Belupo, Koprivnica, Croatia). Intrasulcular incisions and vertical releasing incisions were done with a scalpel blade No. 15 to expose the site of the extraction. All tooth extractions were performed with atraumatic instruments. In the case of multi-rooted teeth, root separation was performed before extraction to avoid damage to hard and soft tissue. After extraction, the surgeon performed curettage of the alveolus to remove granulation tissue, following which patients were randomized into two groups (see again Figure 1). Blinding was not possible because the test group received the injection material in a syringe, while in the control group granulated biomaterial was used. The free web interface https://www.randomizer.org/ (accessed 10 October 2020.) was used to randomize patients. The test group was treated with IBCP composed of a water-based gel with nano-hydroxyapatite particles and biphasic calcium phosphate granules (60% HA and 40% β–TCP; the granules account for 16.5% of the paste; maxresorb^®^ inject, botiss biomaterials, Zossen, Germany). In the control group, a BX (cerabone^®^, botiss biomaterials, Zossen, Germany) was placed in the socket following extraction. In both groups, the graft material was gently pressed into the socket and covered with a resorbable membrane made of porcine collagen (collprotect^®^ membrane, botiss biomaterials, Zossen, Germany) (Figure 2 and Figure 3). Patients were instructed to continue antibiotic therapy for 7 days postoperatively (amoxicillin/clavulanic acid 2 × 1000 mg per day or clindamycin 2 × 600 mg per day) and to maintain adequate oral hygiene.

### 2.3. Re-Entry Procedure and Biopsy Harvesting

The second phase was performed six months later. The preoperative protocol was the same. The full-thickness mucoperiosteal flap was elevated, and a bone biopsy was taken from the central part of the preexisting defect using a trephine drill (2.5 mm inner diameter; Ustomed^®^ Instrumente, Tuttlingen, Germany). The trephine drill was then stored in 4% formaldehyde before proceeding to the histologic analysis. After biopsy collection, the oral surgeon placed the dental implant using a standardized implant set. Patients were instructed not to brush their teeth around the surgical site and to rinse their mouth twice daily with chlorhexidine mouthwash. 

### 2.4. Biopsy Preparation and Histologic Evaluation

Trephine drills containing the biopsies were fixed in 4% formaldehyde solution for two weeks. After fixation, the specimens were placed in ethylenediaminetetraacetic acid (Decalcifier soft^®^, Solvagreen^®^, Karlsruhe, Austria) for decalcification and then placed in the tissue processor (MTP, SLEE Medical GmbH, Mainz, Germany). The biopsies were embedded in paraffin wax using the modular unit (MPS/P, SLEE Medical GmbH, Mainz, Germany), and sectioned using a microtome (CUT 4062, SLEE Medical GmbG, Mainz, Germany). The slides were then stained with hematoxylin-eosin and examined under a light microscope (Leica DMRB, Leica Microsystems GmbH, Wetzlar, Germany) in conjunction with a video camera (Axio Imager M2, Zeiss, Oberkochen, Germany). Pathohistological assessment was performed by a single examiner (Ž.P.K.). Photomicrographs were taken under 10× objective magnification and transferred to ImageJ software (NIH, https://imagej.nih.gov/ij/, accessed on 14 November 2021.) for histomorphometric analysis. For histomorphometric analysis, three sections were taken from the central part of each biopsy, separated by 50 μm and measurements of the total area, area of bone tissue, and residual biomaterial area were undertaken and transferred to a Microsoft Excel spreadsheet. To obtain the soft tissue area, the area of bone tissue and remaining biomaterial was subtracted from the total area. Finally, the mean values based on the analysis of the sections were converted to volume percentages of newly formed bone (NB), residual biomaterial (BM), and soft tissue (ST). 

### 2.5. Sample Size and Statistical Analysis

For a significance level α of 0.05, a test power of 80%, an expected standard deviation of the predicted result of 10, and an equivalence limit of d = 10, of at least 18 subjects per group should be included in the study. Statistical analysis was performed using the statistical program (SPSS 25.0, SPSS Inc., Chicago, IL, USA). Quantitative data were expressed as mean and standard deviation. The Mann—Whitney U test was used to compare histomorphometric percentages of NB, residual BM, and ST between the test and control groups. All *p*-values less than 0.05 are considered significant.

## 3. Results

### 3.1. Clinical Observations

A total of 41 patients were included in the study. The reason for extraction was untreatable chronic periodontitis in 35 patients and deep crown or root fracture in six patients. One participant in the test group dropped out of the study because the participant chose to postpone implant therapy for more than six months. Finally, biopsies were harvested from 20 patients in the test group and 20 in the control group (see again Figure 1). Patient population characteristics are shown in Table 1. The distribution of extraction sites is shown in Table 2. Each participant had only one extraction site.

The IBCP was easy to handle and insert into the extraction socket. The healing phase was uneventful. In total, five patients in the test group and two in the control group reported pain sensations and small edema. No membrane exposure was noted during early postoperative checks, which took place on the first, seventh, and tenth days postoperatively. Leakage of the bone substitute was not observed in any group. Later controls took place on the 30th, 60th, and 90th postoperative days. On the 90th postoperative day, wound healing was observed as complete closure of the oral mucosa at the extraction site in all participants.

### 3.2. Qualitative Histologic Analysis

**Figure 4:**
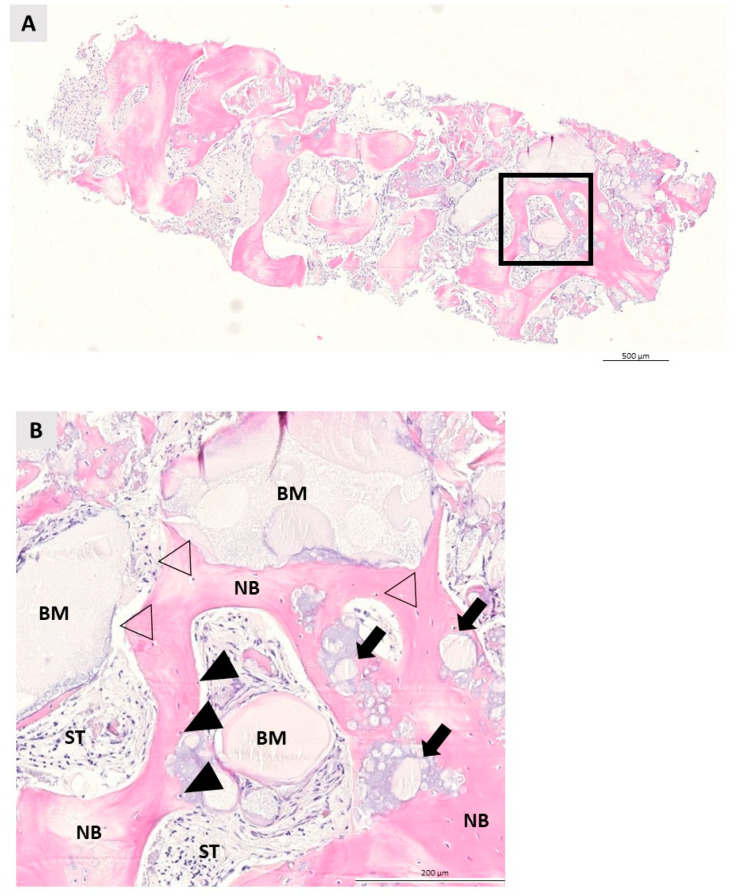
Representative specimen of a bone biopsy taken six months after augmentation with maxresorb^®^ inject. (**A**) Longitudinal section through the specimen showing the new bone formation and the IBCP granules integrated into it. The square marks the area of interest in the figure below (hematoxylin-eosin, 10× magnification). (**B**) Details of the area of interest. The IBCP (biomaterial = BM) is in direct contact with newly formed bone (NB) and soft tissue (ST). Formation of NB begins at the boundary between the IBCP and the defect. The cells in the calcified bone matrix are osteocytes (no filling triangles), whereas osteoblasts (black filling triangles), their precursor, were detected at the boundary between IBCP and NB. Note the remaining IBCP granules (black filling triangles) integrated into the NB. No inflammatory tissue reaction was observed (hematoxylin-eosin, 20× magnification).

**Figure 5:**
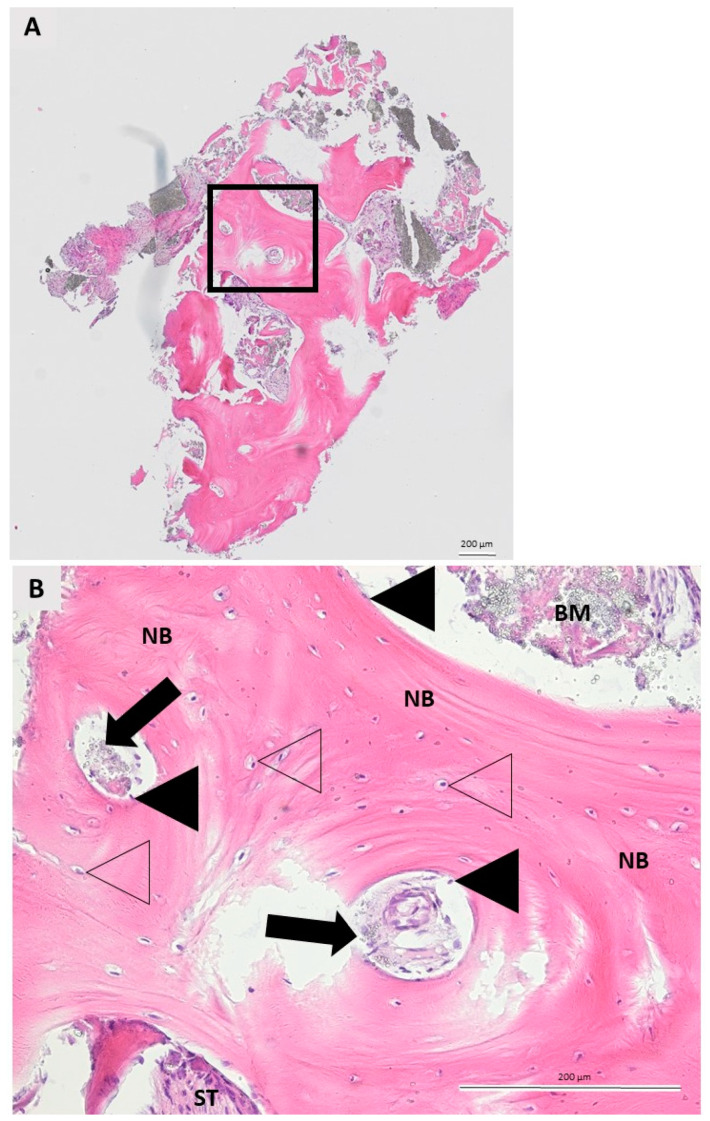
Representative specimen of a bone biopsy taken six months after augmentation with cerabone^®^. (**A**) Longitudinal section of specimen shows newly formed bone (NB) and connective soft tissue (ST) surrounding the remaining biomaterial. The square marks the region of interest (hematoxylin-eosin, 10× magnification). (**B**) A photomicrograph showing details of the selected area. The NB is rich in entrapped osteocytes (no filling triangles), whereas osteoblasts (black filling triangles) are seen at the interface between the remaining biomaterial (BM) and NB. Small, residual particles of BX (filling arrows) are surrounded by NB. The ST area contains mainly fibroblasts. There are no signs of inflammatory tissue reaction to the implanted BM (hematoxylin-eosin, 20× magnification).

Qualitative histologic analysis was performed for each specimen in both groups. NB was clearly visible in both groups, indicating successful bone growth from the apical to the coronal segment of the specimens. The residual BM was surrounded by new bone and non-mineralized tissue and was easily recognizable in both groups due to its irregular appearance. Bone growth began in both groups at the boundaries where the biomaterial and pristine bone were in direct contact. The NB showed a regular lamellar structure with osteocytes enclosed within. Osteoblasts, active cells indicative of bone remodeling, were detected at the contact between BM and NB. The non-mineralized ST area was full of fibroblasts. After 6 months of healing, multinucleated giant cells (MNGs), indicative of a foreign body reaction, were not detected in either group. No inflammatory tissue reaction was observed in either the control or test group, indicating the biocompatibility of the bone graft with the surrounding tissue (see Figure 4 and Figure 5).

### 3.3. Quantitative Histologic Analysis

Quantitative histologic analysis showed comparable results in terms of the percentage of NB and residual BM in both groups. Socket preservation with IBCP resulted in an average percentage of 26.47 ± 14.71% of NB and 13.1 ± 14.07% of residual BM. A moderately higher percentage of NB and residual BM was observed in the control group, with a mean of 30.47 ± 16.39% and 17.89 ± 11.81%, respectively. However, no significant difference was observed between the test and control groups in the percentage of NB and residual BM (*p* > 0.05, Mann—Whitney U test). Statistical significance between the groups was found in results of soft tissue percentage (IBCP: 60.43 ± 12.73% vs. BX: 51.64 ± 14.63%, *p* = 0.046, Mann—Whitney U Test) (Table 3). 

## 4. Discussion

Socket preservation prevents alveolar ridge volume loss after tooth extraction. Different grafting materials are used to achieve optimal alveolar ridge volume after tooth extraction, which is essential for an optimal esthetic and functional outcome of implant-prosthetic rehabilitation. Although autogenous bone is considered the gold standard, focus is being placed on different graft materials, primarily to avoid the complications associated with the use of autogenous bone. In this randomized controlled clinical study, we evaluated the qualitative and quantitative histological changes in bone biopsies harvested six months after socket preservation according to GBR principles with IBCP (maxresorb^®^ inject) and BX (cerabone^®^). As far as we know, this is the first published human histologic study on the use of IBCP, composed of a water-based gel with nano-hydroxyapatite particles and biphasic calcium phosphate granules (60% HA and 40% β–TCP) in socket preservation, and its comparison with BX. Both biomaterials showed good integration into the NB and no inflammatory reaction was observed in the tissue. Furthermore, both biomaterials showed comparable histomorphometric results with regard to new bone formation (IBCP: 26.47 ± 14.72% and BX: 30.47 ± 16.39%, *p* = 0.659, Mann—Whitney U Test).

BCP ceramics combine good properties of HA and β-TCP. The resorption rate of BCPs can be slow or faster depending on the HA and β-TCP ratio. A comparative histological evaluation of BCPs with different ratios of HA and β-TCP in socket preservation showed that BCP composed of HA (60.28%) and β-TCP (39.72%) had the highest percentage of NB after 6 months of healing [35]. The IBCP used in this study has granules with almost the same HA and β-TCP ratio. During bone healing, β-TCP is rapidly resorbed, and volume stability of the graft until it is replaced by new bone is provided by a high proportion of HA, which is a more stable and less resorbable component of BCP [35,36]. These observations are consistent with our qualitative and quantitative histologic findings that 6 months after socket preservation residual particles of biomaterial (IBCP: 13.1 ± 14.07%, BX: 17.89 ± 11.81%, *p* = 0.121, Mann—Whitney U Test) were integrated into the NB in both groups. 

The use of IBCP in dental indications is poorly investigated. An in vitro study has shown that IBCP can serve as an rh-BMP9 carrier and that it is easy to handle [37]. An animal study showed that IBCP allows fast tissue influx between biomaterial granules from day 10 [36]. To our knowledge, only three human studies have been published on the performance of IBCP. The first of these studies was published in 2007. by Weiss et al. and was the first report of the clinical results of IBCP. Only three biopsies were histologically evaluated three years after augmentation. The BCP granules were in close contact with the bone tissue, suggesting that the biomaterial supports bone growth [38]. This finding is consistent with the qualitative histologic findings of IBCP used in our study. However, Weiss et al. analysed only three biopsies taken three years after socket preservation. This is a long time period considering that most histologic analysis studies have been performed on biopsies taken after 4 to 9 months of healing. The study by Lorenz et al. [39] focused on the regenerative potential of IBCP based on β-TCP and hyaluronic acid in socket preservation. Biopsies were taken 4 months after augmentation, and histomorphometric analysis showed new bone formation of 44.92 ± 5.16%. In our study, new bone formation was lower, but this could be due to the different timing of harvest and composition of the biomaterial, as β-TCP tends to resorb rapidly. Recently, the clinical results of sinus floor elevation with BCP (60% HA and 40% β-TCP) coated with a layer of polylactic-co-glycolic acid (PLGA) prepared in a plastic syringe and their comparison with BX were published. The aforementioned biomaterial showed the same performance in terms of sinus floor elevation, but was easier to handle and apply than BX [40]. We made the same observation regarding the handling properties of the IBCP used in the current study. Due to its viscosity, the biomaterial was easily applied into the extraction sockets and filled them completely. In addition, the investigated biomaterial in this study was also pre-filled in a sterilized plastic syringe, which expedited the surgical procedure and reduced the treatment burden. 

Previous comparative histologic studies of BX and BCP were mostly based on the maxillary sinus model [41,42,43,44,45,46,47,48,49]. In the study by Oh et al. [41], histomorphometric analysis 6 months after sinus floor augmentation showed no significant difference in histomorphometric results between the BX and BCP group. In a similar randomized clinical trial by Kraus et al. [43], it was published that the BX and BCP groups had comparable new bone formation, but the BX group had a significantly higher percentage of residual biomaterial and a lower percentage of soft tissue than the BCP group. In the study by Cordaro et al. [47] the same conclusions were reported. Interestingly, we observed similar results in our study, where the percentage of soft tissue showed a statistically significant difference between groups (IBCP: 60.43 ± 12.73% vs. BX: 51.64 ± 14.63%, *p* = 0.046, Mann—Whitney U Test). Although the difference is small, we can say that it is borderline, but still it is statistically significant. The question of whether or not this significance has clinical implications should be the focus of future research.

All in all, the above results regarding the comparison of new bone formation between BCP and BX are in agreement with our findings 6 months after socket preservation. However, we must point out that because of the different clinical indications, the different times at which the biopsies were harvested, the material composition (HA and β-TCP ratio), and the fact that we used an injectable form of BCP, an objective comparison with the aforementioned studies is limited. 

Among all bone grafts, cerabone^®^ was selected as biomaterial in the control group. Cerabone^®^ has been well studied to date in both preclinical animal studies [50,51,52,53,54,55,56] and human clinical trials [57,58,59]. Socket preservation using cerabone^®^ has proven to be a reliable method to minimize the loss of bone volume at the extraction site [57]. In the current study, cerabone^®^ has shown new bone formation of 30.47 ± 16.39% 6 months after socket preservation. A previous histomorphometric analysis of biopsies collected after augmentation of the sinus floor with cerabone^®^ in two postoperative periods, early (mean: 5.73 ± 0.44 months) and late (mean: 8.68 ± 1.76 months), showed new bone formation in the early group of 22.77% ± 5.89% and 26.15% ± 11.18% in the late group [58]. Our results suggest higher new bone formation, but it should be considered that we evaluated our histomorphometric results 6 months after socket preservation. Overall, cerabone^®^ shows promotion of osteoconduction, complete integration into the NB, and a slow resorption rate as well as good handling properties. It is known that cerabone^®^ and Bio-Oss™ (Geistlich Biomaterials, Wolhusen, Switzerland) are two of the most commonly used BXs [9]. Bio-Oss™ is the most investigated BX, with a similar performance to autogenous bone [60]. A quantitative histological comparison between cerabone^®^ and Bio-Oss™ showed comparable results in terms of the mean percentage of new bone formation, with no significant difference between the groups [59]. In addition, a retrospective study published by Mahesh et al. [61] showed comparable results between these two biomaterials in terms of new bone formation and tissue response 6 months after sinus floor augmentation. All in all, it can be concluded that cerabone^®^ is a good and reliable biomaterial whose performance is consistent with that of Bio-Oss™, and both of them can challenge the gold standard in bone augmentation–autogenous bone.

It should be noted that the limitation of this study is the lack of a negative control group consisting of sockets with no intervention in healing. Nevertheless, socket preservation minimizes bone resorption compared with extraction without intervention [62]. Therefore, we did not want to include a negative control group in this study primarily for ethical reasons toward patients who deserve the best treatment option. 

Recent developments in the field of BCPs report novel modifications, such as incorporation of bioactive agents and tuning of composition, porosity, and roughness, which may represent the future for new generation materials for bone remodeling [63].

To summarize, both IBCP and BX provide comparable histomorphometric results for newly formed bone. Based on qualitative histologic findings we can conclude that both biomaterials have good osteoconductive properties and biocompatibility. The higher percentage of soft tissue in the IBCP group and its clinical relevance should be the focus of future research, as well as the regenerative potential of IBCP in larger alveolar defects.

## Figures and Tables

**Table 1:** Demographic data of participants.

	IBCP ^1^	BX ^2^
Gender		
Female	13 (65%)	12 (60%)
Male	7 (35%)	8 (40%)
*n*	20	20
Age (years)		
Mean	37.9	35.9
SD	12	11.1
Min	19	19
Max	59	55

^1^ Injectable biphasic calcium phosphate, ^2^ Bovine xenograft.

**Table 2:** Distribution of extraction sites.

	Incisor	Canine	Premolar	Molar	Total
Mandible IBCP ^1^	4	1	2	5	12
Maxilla IBCP ^1^	2	1	3	2	8
Mandible BX ^2^	2	1	2	6	11
Maxilla BX ^2^	2	0	3	4	9
Total	10	3	10	17	40

^1^ Injectable biphasic calcium phosphate, ^2^ Bovine xenograft.

**Table 3:** Histomorphometrical results.

	Newly Formed Bone (NB)	Residual Biomaterial (BM)	Soft Tissue (ST)
IBCP ^1^	26.47 ± 14.72%	13.1 ± 14.07%	60.43 ± 12.73%
BX ^2^	30.47 ± 16.39%	17.89 ± 11.81%	51.64 ± 14.63%
*p*-value *	0.659	0.121	0.046

^1^ Injectable biphasic calcium phosphate, ^2^ Bovine xenograft; * Mann—Whitney U Test.

## Data Availability

The data presented in this article are available on request from the corresponding author.
